# Treatment of adults with intracranial hemorrhage on apixaban or rivaroxaban with prothrombin complex concentrate products

**DOI:** 10.1007/s11239-020-02154-z

**Published:** 2020-06-04

**Authors:** Renee Castillo, Alissa Chan, Steven Atallah, Katrina Derry, Mark Baje, Lara L. Zimmermann, Ryan Martin, Leonid Groysman, Sara Stern-Nezer, Anush Minokadeh, Alan Nova, WanTing Huang, William Cang, Kendra Schomer

**Affiliations:** 1 Department of Pharmacy, University of California, Irvine Health, Orange, CA USA; 2 Department of Neurology, University of California, Irvine Health, Orange, CA USA; 3 Department of Pharmacy, University of California San Diego Health, San Diego, CA USA; 4 Department of Critical Care, University of California San Diego Health, San Diego, CA USA; 5 Department of Pharmacy, University of California Davis Health, Sacramento, CA USA; 6 Department of Neurological Surgery and Neurology, University of California Davis Health, Sacramento, CA USA

**Keywords:** Prothrombin complex concentrates, Intracranial hemorrhages, Hematomas, Factor xa inhibitors, Direct oral anticoagulants, Hemostatics

## Abstract

To analyze the efficacy and safety of activated prothrombin complex concentrates (aPCC) and four-factor prothrombin complex concentrates (4F-PCC) to prevent hematoma expansion in patients taking apixaban or rivaroxaban with intracranial hemorrhage (ICH). In this multicenter, retrospective study, sixty-seven ICH patients who received aPCC or 4F-PCC for known use of apixaban or rivaroxaban between February 2014 and September 2018 were included. The primary outcome was the percentage of patients who achieved excellent/good or poor hemostasis after administration of aPCC or 4F-PCC. Secondary outcomes included hospital mortality, thromboembolic events during admission, and transfusion requirements. Excellent/good hemostasis was achieved in 87% of aPCC patients, 89% of low-dose 4F-PCC [< 30 units per kilogram (kg)], and 89% of high-dose 4F-PCC (≥ 30 units per kg). There were no significant differences in excellent/good or poor hemostatic efficacy (p = 0.362). No differences were identified in transfusions 6 h prior (p = 0.087) or 12 h after (p = 0.178) the reversal agent. Mortality occurred in five patients, with no differences among the groups (p = 0.838). There were no inpatient thromboembolic events. Both aPCC and 4F-PCC appear safe and equally associated with hematoma stability in patients taking apixaban or rivaroxaban who present with ICH. Prospective studies are needed to identify a superior reversal agent when comparing andexanet alfa to hospital standard of care (4F-PCC or aPCC) and to further explore the optimal dosing strategy for patients with ICH associated with apixaban or rivaroxaban use.

**Electronic supplementary material:**

The online version of this article (10.1007/s11239-020-02154-z) contains supplementary material, which is available to authorized users.

## Highlights

- This study sought to analyze the efficacy and safety of aPCC and 4F-PCC used to prevent hematoma expansion in patients taking apixaban or rivaroxaban with ICH.
- The primary outcome was the percentage of patients with ICH who achieved excellent/good or poor hemostasis after administration of 4F-PCC or aPCC, determined by physician review of serial CT scans within 12 h of reversal agent administration.
- Both aPCC and 4F-PCC appear safe and equally associated with hematoma stability in patients taking apixaban or rivaroxaban who present with ICH after trauma or spontaneous ICH.
- Prospective studies are needed to identify a superior reversal agent when comparing andexanet alfa to hospital standard of care (4F-PCC or aPCC) and to further explore the optimal dosing strategy for patients with ICH associated with apixaban or rivaroxaban use.

## Introduction

The use of Factor Xa (FXa) inhibitors have increased due to the lower incidences of intracranial bleeding, ease of monitoring, and fewer dietary restrictions relative to vitamin K antagonists [1–5]. There is still a paucity of data for the management of life-threatening bleeding, such as intracranial hemorrhage (ICH). Before the FDA approval of andexanet alfa, agents utilized in ICH associated with FXa inhibitor use were four-factor prothrombin complex concentrate (4F-PCC) and activated prothrombin complex concentrate (aPCC).

The Neurocritical Care/Society of Critical Care Medicine (NCS/SCCM) guidelines recommend either 4F-PCC or aPCC at a dose of 50 units per kilogram (kg) for a life-threatening bleed, such as an ICH [6, 7]. NCS/SCCM favors the use of 4F-PCC or aPCC as these agents correct anti-factor Xa-associated coagulopathy and coagulation parameters. These recommendations were published prior to the approval of andexanet alfa. The 50 units per kg dose was based on studies conducted in healthy humans and in animal models of hemorrhagic injury [8, 9]. After the publication of the NCS/SCCM guidelines, other trials have demonstrated achievement of effective bleeding control when prothrombin complex concentrates were used for management of major bleeding, however, objective assessment of hemostatic efficacy is limited within these studies [10–16].

Recently, a new antidote andexanet alfa, recombinant modified human Factor Xa, received accelerated approval from the Food and Drug Administration (FDA) for the reversal of apixaban and rivaroxaban for life-threatening or uncontrolled bleeding [17]. However, cost, thrombotic risk, and concern for rebound bleeding have prevented wide adoption of andexanet alfa.

This multicenter, retrospective study sought to analyze the hemostatic efficacy and safety of treatment for FXa inhibitor associated coagulopathy with aPCC or 4F-PCC in patients presenting with ICH. We hypothesized that treatment with either < 30 units per kg of 4F-PCC,  ≥ 30 units per kg of 4F-PCC, or a range of 8–50 units per kg of aPCC would achieve effective hemostasis when used in patients with ICH associated with apixaban and rivaroxaban use [6, 7, 10–16].

## Materials and methods

### Study design and oversight

This study was approved by the institutional review board at each facility. Data was extracted from the electronic medical record (EMR) and reviewed by a pharmacist. Within each trauma center, two intensivist physicians independently reviewed each computed tomography of the head (CTH), calculated ICH volume or thickness, and in patients with spontaneous intraparenchymal hemorrhage (IPH), the ICH score [18]. Institutional guidelines for dosing of 4F-PCC and aPCC and transfusion of blood products were followed by each respective facility at the time of initial management.

### Study population

This retrospective study evaluated patients from February 1, 2014 through September 30, 2018 at three level I trauma centers who were admitted with apixaban- or rivaroxaban-related ICH and treated with at least one dose of 4F-PCC or aPCC. FXa inhibitor use was determined via the home medication list, patient’s family when present, and/or the primary team. Patients were included if they were at least 18 years of age and had a baseline CTH scan prior to and a follow up CTH scan within 12 h of 4F-PCC or aPCC administration. ICH was defined as traumatic or nontraumatic and subclassified as subarachnoid hemorrhage (SAH), IPH, intraventricular hemorrhage (IVH), subdural hematoma (SDH), or epidural hematoma (EDH). Patients were excluded if they had acute on chronic or chronic ICH, underwent major neurosurgical intervention, such as a surgical hematoma evacuation between the baseline and within the 12-h follow-up scan, and/or had an ICH volume ≥ 60 mL. One center exclusively used aPCC at a dose of 8–50 units per kg at the discretion of the treating team. The other two institutions used 4F-PCC at a range of 25 or 50 units per kg. Although each respective institution had a dosing guideline, ordering providers could adjust doses. After reviewing the 4F-PCC data, the patients were divided into two different dosing schemes, less than 30 units per kg and ≥ 30 units per kg. The patients were then analyzed in three groups: < 30 units per kg of 4F-PCC, ≥ 30 units per kg of 4F-PCC, and 8–50 units per kg of aPCC.

### Data collection and outcomes

Administration of agents that affect hemostasis prior to admission (e.g. antiplatelets) and during initial inpatient management (e.g. desmopressin, vitamin K) were recorded. Initial and discharge Glasgow Coma Scale (GCS), ICH score, and length of hospital stay were also collected. We utilized a modified rating system adopted from Sarode et al. and Connolly et al. to determine hemostatic efficacy (Table 1) [19–21].

**Table 1:** Rating system for hemostatic efficacy [16, 17]

Bleed type	Excellent	Good	Poor
Intraparenchymal hematoma	< 20% increase in hematoma volume compared to baseline on a repeat CT scan performed within 12 h of reversal agent administration	> 20% but < 35% increase in hematoma volume using the most dense area on a repeat CT scan performed within 12 h of reversal agent administration	> 35% increase in hematoma volume using the most dense area on a repeat CT scan performed within 12 h of reversal agent administration
Subarachnoid bleed, subdural hematoma	< 20% increase in maximum thickness compared to baseline on a repeat CT scan performed within 12 h of reversal agent administration	> 20% but < 35% increase in maximum thickness using the most dense area on a repeat CT scan performed within 12 h of reversal agent administration	> 35% increase in maximum thickness using the most dense area on a repeat CT scan performed within 12 h of reversal agent administration

The primary outcome assessed was the percentage of ICH patients who achieved excellent/good or poor hemostasis after administration of 4F-PCC or aPCC (Table 1). Percentage of increase in volume or thickness of ICH was calculated by comparing the baseline CTH scans to the CTH scan within 12 h of 4F-PCC or aPCC administration. Baseline CTH scans were defined as the CTH scan prior to 4F-PCC or aPCC administration. Follow-up scans were the CTH scan closest to and within 12 h of 4F-PCC or aPCC administration. Two physician investigators at each facility independently calculated each IPH volume using the ABC/2 method [22]. The size of SAH, SDH, and EDH were defined as the largest measured thickness on axial images. If multiple types of ICH were present, the largest was assessed for the primary outcome. An adjudication process was conducted for any difference in physician readings. There was no minimum duration required between factor product administration and repeat CTH.

Secondary outcomes included mortality during admission, thromboembolic events documented during admission, recorded transfusion requirements, and discharge disposition. Thromboembolic events were defined as type 1 myocardial infarction, pulmonary embolism, stroke, or deep vein thrombosis. Mortality and thromboembolic events were collected through chart review and discharge summaries. We analyzed low-dose (< 30 units per kg) 4F-PCC, high-dose (≥ 30 units per kg) 4F-PCC, and aPCC for all outcomes.

### Statistical analysis

Continuous variables are presented as mean ± standard deviation or median and interquartile range. Binomial data is presented as proportions or percentages and compared using the chi square test. A p value of less than 0.05 was considered statistically significant with a 2-sided test. All statistical analysis was performed using STATA IC 14.

## Results

**Fig. 1:**
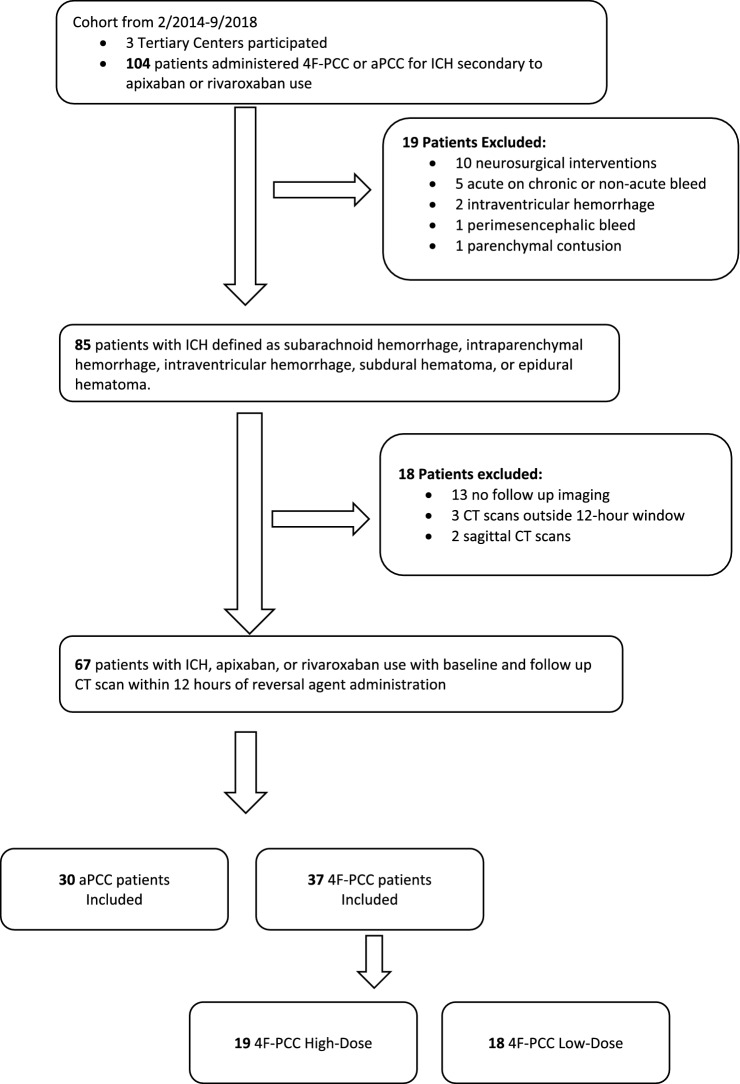
Study flow diagram

One-hundred and four patients with ICH related to apixaban or rivaroxaban use were reviewed (Fig. 1). We excluded ten patients who received neurosurgical interventions. Nine patients did not have a qualifying type of ICH. Sixteen patients did not have CTH scans or had follow up scans that were greater than 12 h from administration of the reversal agent. Sixty-seven ICH patients were included with a baseline and a follow up CTH scan within 12 h of reversal agent administration. Table 2 summarizes the baseline characteristics.

**Table 2:** Baseline characteristics and initial management of study population

Characteristics	aPCC (n = 30)	Low-dose 4F-PCC (n = 18)	High-dose 4F-PCC (n = 19)	p value
Age (years), mean ± SD	74 ± 14	81 ± 11	80 ± 11	0.110
Female, no (%)	14 (47%)	6 (33%)	9 (47%)	0.608
Weight (kg), mean ± SD	80.0 ± 18.7	80.6 ± 16.8	75.6 ± 19.3	0.700
GCS upon admission, median [IQR]	15 [6–15]	15 [6–15]	14 [3–15]	
GCS at discharge, median [IQR]	15 [6–15]	15 [6–15]	14 [3–15]	
Total hospital stay (days), median [IQR]	4 [1–42]	5 [2–26]	5 [2–65]	0.329
PCC dose (units per kg), mean ± SD	19.1 ± 8.0	24.6 ± 2.74	48.8 ± 4.8	
Time from presentation to reversal administration (hrs), median [IQR]	2.7 [0.8–47.0]	3.1 [0.74–20.5]	2.6 [0.7–11.1]	0.430
Direct anticoagulant				
Apixaban, no (%)	14 (47%)	6 (33%)	9 (47%)	0.608
Rivaroxaban, no (%)	16 (53%)	12 (67%)	10 (53%)	0.608
Anticoagulant indication				
Atrial fibrillation	20 (67%)	14 (78%)	18 (95%)	0.72
Pulmonary embolism	3 (10%)	1 (6%)	N/A	0.354
Deep vein thrombosis	1 (3%)	1 (6%)	1 (5%)	0.919
Stroke	1 (3%)	1 (6%)	1 (5%)	0.919
Multiple indications (≥ 1 above)	3 (10%)	N/A	N/A	0.144
Mechanism of ICH				
Spontaneous, no (%)	10 (33%)	8 (44%)	5 (26%)	0.504
Traumatic, no (%)	20 (67%)	10 (56%)	14 (74%)	0.504
Types of ICH				
Subarachnoid hemorrhage, no (%)	2 (7%)	5 (28%)	3 (16%)	0.057
Intraparenchymal hemorrhage, no (%)	8 (27%)	7 (39%)	4 (21%)	0.619
Intraventricular hemorrhage, no (%)	0	1 (5.5%)	0	0.251
Subdural hematoma, no (%)	10 (33%)	4 (22%)	11 (58%)	0.205
Multiple^a^, no (%)	8 (27%)	1 (5.5%)	1 (5%)	0.714
Size of ICH				
Hematoma volume^b^, mean	13.8 ± 11.9	8.4 ± 12.1	22.0 ± 18.0	0.505
Hematoma maximum thickness^c^, mean	9.5 ± 4.3	27.2 ± 16.1	9.4 ± 4.3	0.0003
ICH score, median	1	2	3	0.040
Outpatient medications given affecting hemostasis				
Aspirin, no (%)	11 (37%)	2 (11%)	3 (16%)	0.082
Clopidogrel, no (%)	3 (10%)	0 (0%)	2 (11%)	0.370
Inpatient medications given affecting hemostasis				
Desmopressin, no (%)	5 (17%)	0 (0%)	0 (0%)	0.036
Tranexamic acid, no (%)	0 (0%)	0 (0%)	2 (11%)	0.074
Transfusion requirements				
Fresh frozen plasma				
6 h before, no (%)	0 (0%)	3 (17%)	2 (11%)	0.087
12 h within, no (%)	0 (0%)	2 (11%)	2 (11%)	0.178

*GCS* glasgow coma scale, *IQR* interquartile range, *SD* standard deviation

^a^Multiple is defined as multiple types of ICH

^b^Hematoma volume is measured in cm^3^

^c^Baseline subdural hematoma maximum thickness measured in mm

The mean age of the study population was 77 years and 43% were female. Thirty-eight patients (57%) were on rivaroxaban and 29 patients (43%) were on apixaban. The mechanism of ICH was predominantly traumatic (70%). Subdural hematoma (30%) and intraparenchymal hemorrhage (30%) were the most common type of ICH analyzed. Upon hospital presentation and initial management, patients had a median GCS of 15 for both aPCC and low-dose 4F-PCC and a GCS of 14 for high-dose 4F-PCC. The median ICH score for patients with a spontaneous IPH was 1 in aPCC, 2 in low-dose 4F-PCC and 3 in high-dose 4F-PCC.

### Treatment with reversal agent

Mean doses administered within the aPCC, low-dose and high-dose 4F-PCC are summarized in Table 2. The median [IQR] time (hours) from hospital presentation to reversal agent administration was 2.7 [0.8–47.0] with aPCC, 3.1 [0.74–20.5] with low-dose 4F-PCC, and 2.6 [0.7–11.1] with high-dose 4F-PCC. A second dose of 4F-PCC was administered within 24 h in one patient within the low-dose 4F-PCC group.

### Additional medications and blood products administered affecting hemostasis

Documentation of other medications that affect hemostasis administered during initial inpatient management are listed in Table 2. There were no significant differences in use of antiplatelets prior to admission. During initial admission, two patients with high-dose 4F-PCC received tranexamic acid and five patients received desmopressin in the aPCC group. No aPCC patients received fresh frozen plasma (FFP) while 17% of low-dose 4F-PCC patients and 11% of high-dose 4F-PCC patients received FFP within 6 h prior to reversal. Within 12 h after treatment administration, 11% in the low and high-dose 4F-PCC groups received FFP.

### Primary outcome: hemostatic efficacy

**Fig. 2:**
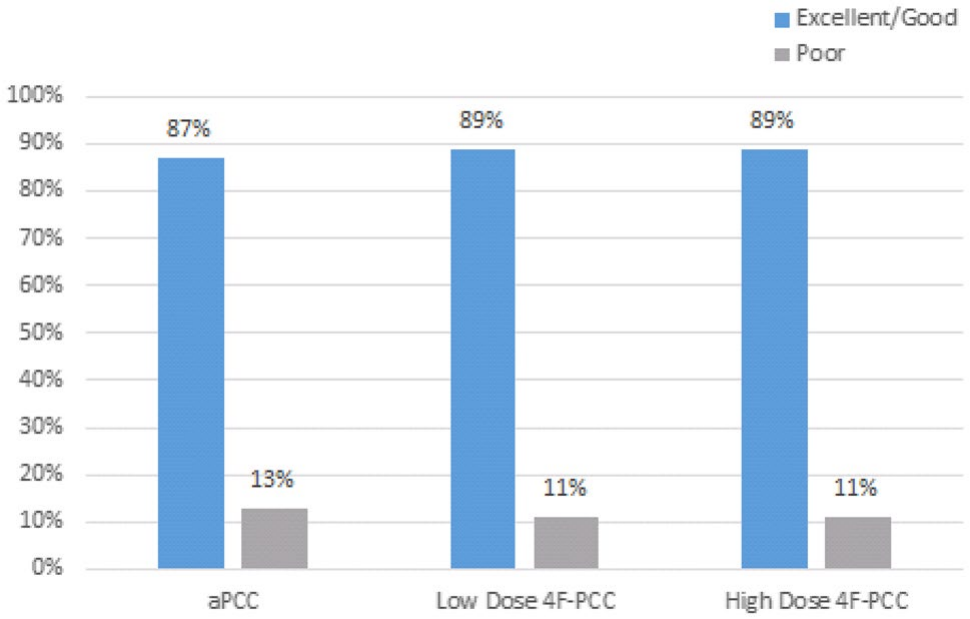
Overall Hemostatic Efficacy (p = 0.362) –bar graph conveying hemostatic efficacy in aPCC, low and high-dose 4F-PCC. Blue demonstrates excellent and good and gray demonstrates poor hemostatic efficacy

After final adjudication, the hemostatic efficacy of the treatment agents for ICH patients with apixaban or rivaroxaban use were deemed excellent/good in 87% that were treated with aPCC and 89% in both the low and high-dose 4F-PCC (Fig. 2, p = 0.362). Further breakdown of excellent and good hemostasis demonstrated excellent hemostasis in 83% treated with aPCC, 67% with low-dose 4F-PCC, and 79% with high-dose 4F-PCC. Good hemostatic efficacy was achieved in 3% with aPCC, 22% with low-dose 4F-PCC, and 11% in high-dose 4F-PCC. Poor hemostatic efficacy was found in 13% in aPCC, 11% in low-dose 4F-PCC, and 11% in high-dose 4F-PCC. Across all three groups, there were no significant differences in hemostatic efficacy. When comparing aPCC and 4F-PCC, 87% and 89% achieved excellent or good hemostatic efficacy, respectively (p = 0.954).

### Secondary outcomes and safety

A safety analysis was performed for included patients. No thromboembolic events occurred within 30 days of admission. Death occurred in a total of 5 patients, with no statistically significant differences in inpatient mortality across all groups (p = 0.838). Four patients with inpatient mortality had excellent or good hemostatic efficacy while one patient had poor efficacy. There were no significant differences in transfusion requirements 6 h prior (p = 0.087) and 12 h after (p = 0.178) administration of the reversal agent across three groups. The majority of patients, regardless of reversal agent administered, were discharged to either a skilled nursing facility (43%) or home (45%) (Online Resource 1, p = 0.221).

## Discussion

Intracranial hemorrhage associated with FXa use is a medical emergency [23]. Several trials have demonstrated effective bleeding control in the majority of patients with Factor Xa inhibitor associated ICH who received reversal agents [10–16]. The UPRATE study demonstrated effective hemostasis in 72% of their ICH subpopulation treated with a median dose of 26.7 units per kg [11]. Schulman et al. identified effective hemostasis in 83% of 36 ICH patients with a standard dose of 2000 units (26.4 units per kg based on 70 kg patient) [12]. Our study demonstrated a higher percentage of excellent/good hemostasis in comparison to prior trials. Although we were unable to confirm the last dose of factor Xa inhibitor in this study, the shorter time from admission to administration of treatment with aPCC (2.7 h) or 4F-PCC (3.1 and 2.6 h) possibly contributed to improvements in hemostatic efficacy compared to other trials.

We found a relatively low rate of mortality, 7%, when compared to other studies [10–16, 20]. Additionally, our study supports the use of lower doses as it demonstrated comparable hemostatic efficacy to higher 4F-PCC or aPCC dosing. This supports the findings of more current trials, utilizing this low-dose strategy [10–16].

Andexanet alfa was approved by the FDA in 2018 as the first antidote for apixaban- and rivaroxaban-treated patients with a life-threatening or uncontrolled bleeding [17, 20]. The ANNEXA-4 trial revealed the antidote’s ability to acutely lower apixaban and rivaroxaban anti-Xa levels and found 80% of ICH patients achieved excellent or good hemostasis. However, the trial had a thromboembolic event frequency of 10%, resulting in the FDA issuing a boxed warning for increased thromboembolic and ischemic events [17]. Compared to the ANNEXA-4 trial, our study focused only on ICH patients, while the ANNEXA-4 trial had significant enrollment of patients with GI hemorrhage and had more extensive exclusion criteria. Our study demonstrated similar hemostatic efficacy within 12 h and decreased mortality and thrombotic events. The NCS/SCCM guidelines have not been updated on patients with ICH associated with FXa inhibitors since the FDA approval of this antidote. There is an ongoing clinical trial comparing the use of 4F-PCC or aPCC to andexanet alfa (NCT03661528).

Several strengths of this study include a pragmatic ICH population, broad inclusion criteria to mimic clinical practice, and novel comparison. Many previous studies analyzing major bleeding events for patients on apixaban or rivaroxaban have included multiple types of major bleeding, such as gastrointestinal and intramuscular [11, 12, 15, 16]. We did not have exclusion criteria for low GCS scores or expected survival length [19, 20]. Additionally, there are few studies to date that assessed hemostatic efficacy using CTH scans for ICH patients with FXa inhibitor use [11, 12, 15]. We utilized the scale developed by Sarode et al. and the FDA that incorporates more objective measurements, using CTH scans, to determine efficacy [21]. In order to minimize confirmation bias, we had an adjudication process for physician review of CTH scans. To our knowledge, this is the first study to compare aPCC versus 4F-PCC and differences in dosing strategies in exclusively ICH patients.

Several limitations of our study need to be acknowledged. One limitation is the retrospective nature, wherein data collection occurred post-discharge. This limited the availability of certain information such as bleed onset, last anticoagulant administration, functional outcomes, and other factors that may affect hematoma expansion such as blood pressure during initial management. Lack of certainty regarding timing of the last anticoagulant administration could have resulted in overestimation in the rates of excellent/good hemostasis attributable to the reversal agent. However, the inability to confirm exact timing of the last dose of anticoagulants often reflects clinical practice. Additionally, we were only able to identify thromboembolic events or mortality that was documented during the inpatient admission. Similar to other published studies, we did not include a control group thereby limiting our ability to correlate prevention of hematoma expansion as a result of administering reversal agent with improved clinical outcomes such as death or functional recovery [10–16]. We did find similar hemostasis efficacy amongst the different dosing strategies suggesting that there may not be a significant dose response above a certain threshold. The dosing range of 8–50 unit per kg of aPCC could have introduced selection bias. However, of the aPCC patients, only four patients received greater than 25 units per kg. Lower dosing strategy of PCC has been supported in several prior studies [10–16]. Further studies utilizing a lower dosing strategy and placebo group could elicit any potential clinical benefits of using a reversal agent. Generalizability of these findings are limited by the large proportion of mild-moderate traumatic brain injury patients (57%) and the small number of spontaneous bleeds (34%). Two of the three hospitals did not have anti-Xa levels calibrated to apixaban or rivaroxaban; thus, we were not able to use these laboratory values to confirm the presence of the anticoagulant during initial management. Lastly, each of the three hospitals had different guidelines in initial management, such as administration of blood products and time to CT follow up.

## Conclusion

Both aPCC and 4F-PCC appear safe and equally associated with hematoma stability in patients taking apixaban or rivaroxaban who present with ICH. Prospective studies are needed to identify a superior reversal agent when comparing andexanet alfa to hospital standard of care (4F-PCC or aPCC) and to further explore the optimal dosing strategy for patients with ICH associated with apixaban or rivaroxaban use.

## Electronic supplementary material

Below is the link to the electronic supplementary material.

Supplementary file1 (DOCX 14 kb)

## Data Availability

Not applicable.

## Abbreviations

ICH: Intracranial hemorrhage
aPCC: Activated prothrombin complex concentrates
4F-PCC: Four-factor prothrombin complex concentrates
FXa: Factor xa
NCS/SCCM: Neurocritical care/society of critical care medicine
CT: Computed tomography
SD: Standard deviation
IQR: Interquartile range
GCS: Glasgow coma scale
DDAVP: Desmopressin
