# Correction to: Treatment of adults with intracranial hemorrhage on apixaban or rivaroxaban with prothrombin complex concentrate products

**DOI:** 10.1007/s11239-020-02311-4

**Published:** 2020-10-31

**Authors:** Renee Castillo, Alissa Chan, Steven Atallah, Katrina Derry, Mark Baje, Lara L. Zimmermann, Ryan Martin, Leonid Groysman, Sara Stern‑Nezer, Anush Minokadeh, Alan Nova, WanTing Huang, William Cang, Kendra Schomer

**Affiliations:** 1 Department of Pharmacy, University of California, Irvine Health, Orange, CA USA; 2 Department of Neurology, University of California, Irvine Health, Orange, CA USA; 3 Department of Pharmacy, University of California San Diego Health, San Diego, CA USA; 4 Department of Critical Care, University of California San Diego Health, San Diego, CA USA; 5 Department of Pharmacy, University of California Davis Health, Sacramento, CA USA; 6 Department of Neurological Surgery and Neurology, University of California Davis Health, Sacramento, CA USA

## Correction to: Journal of Thrombosis and Thrombolysis 10.1007/s11239-020-02154-z, 10.1007/s11239-020-02197-2

The article “Treatment of adults with intracranial hemorrhage on apixaban or rivaroxaban with prothrombin complex concentrate products”, written by Renee Castillo, Alissa Chan, Steven Atallah, Katrina Derry, Mark Baje, Lara L. Zimmermann, Ryan Martin, Leonid Groysman, Sara Stern-Nezer, Anush Minokadeh, Alan Nova, WanTing Huang, William Cang, Kendra Schomer, was originally published electronically on the publisher’s internet portal on 4 June 2020 without open access. With the author(s)’ decision to opt for Open Choice the copyright of the article changed on 26 October 2020 to © The Author(s) 2020 and the article is forthwith distributed under a Creative Commons Attribution 4.0 International License, which permits use, sharing, adaptation, distribution and reproduction in any medium or format, as long as you give appropriate credit to the original author(s) and the source, provide a link to the Creative Commons licence, and indicate if changes were made. The images or other third party material in this article are included in the article’s Creative Commons licence, unless indicated otherwise in a credit line to the material. If material is not included in the article’s Creative Commons licence and your intended use is not permitted by statutory regulation or exceeds the permitted use, you will need to obtain permission directly from the copyright holder. To view a copy of this licence, visit https://creativecommons.org/licenses/by/4.0.

