# Supplementary material for: Treatment of adults with intracranial hemorrhage on apixaban or rivaroxaban with prothrombin complex concentrate products
Source: J Thromb Thrombolysis. 2020 Jun 4;51(1):151–8. doi: 10.1007/s11239-020-02154-z (PMC7829230; doi:10.1007/s11239-020-02154-z)
Supplement: Supplementary file 1 — Supplementary file1 (DOCX 14 kb) [file 11239_2020_2154_MOESM1_ESM.docx]

TREATMENT OF ADULTS WITH INTRACRANIAL HEMORRHAGE ON APIXABAN OR RIVAROXABAN WITH PROTHROMBIN COMPLEX CONCENTRATE PRODUCTS

Castillo Renee^1a^; Chan Alissa^3a^; Atallah Steven^1a^; Derry Katrina^3a^, Baje Mark^1a^; Zimmermann Lara L^6b^; Martin Ryan^6b^; Groysman Leonid^2b^; Stern-Nezer Sara^2b^; Minokadeh Anush^4b^; Nova Alan^4b^; Huang WanTing^3a^; Cang William^3a^; Schomer Kendra^5a^

Department of Pharmacy^1^ and Department of Neurology^2^, University of California, Irvine Health, Orange, CA, USA; Department of Pharmacy^3^ and Department of Critical Care^4^, University of California San Diego Health, San Diego, CA, USA; Department of Pharmacy^5^ and Department of Neurological Surgery and Neurology^6^, University of California Davis Health, Sacramento, CA, USA; ^a^PharmD; ^b^MD

Corresponding author’s email address: kjschomer@ucdavis.edu

Journal of Thrombosis and Thrombolysis

**Online Resource 1 Patient Discharge Disposition**

| **Characteristics** | **aPCC**  **(n=30)** | **4F-PCC Low Dose**  **(n=18)** | **4F-PCC High Dose**  **(n=19)** |
| --- | --- | --- | --- |
| Skilled Nursing Facility | 10 (33%) | 8(44%) | 11 (58%) |
| Long Term Care | 0 | 0 | 2 (11%) |
| Transfer | 2 (7%) | 0 | 2 (11%) |
| Home | 16 (53%) | 9 (50%) | 5 (26%) |
| Death | 2 (7%) | 1 (6%) | 1 (5% |
